# Combined C-terminal truncation and point mutations in HBx in genotype D1 HBV-associated hepatocellular carcinoma

**DOI:** 10.1186/s13027-026-00771-6

**Published:** 2026-06-18

**Authors:** Miao Yu, Ali Sameer Alkhawaja, Hamidreza Mollaei, Zokirova Fazila, Rizaev Jasur, Elahe Mosayebnejad Roudbaneh, Elham Mosayebnejad Roudbaneh, Kamyar Mazloum Jalali

**Affiliations:** 1 Department of Plastic and Reconstructive Surgery, Heilongjiang Provincial Hospital, Haerbin, 150001 China; 2 Department of Restorative and Esthetic Dentistry, Faculty of Dentistry, The Islamic University, Najaf, Iraq; 3 Department of Microbiology and Medical Virology, School of Medicine, Kerman University of Medical Sciences, Kerman, Iran; 4 Department of Rehabilitation, Sports Medicine and Physical Education, Bukhara State Medical Institute, Bukhara, Uzbekistan; 5 Department of Public Health and Healthcare Management, Rector of Samarkand State Medical University, Samarkand, Uzbekistan; 6 Department of Nursing, School of Allied Medical Sciences, Guilan University of Medical Sciences, Rasht, Iran; 7 Department of Midwifery, School of Allied Medical Sciences, Guilan University of Medical Sciences, Rasht, Iran

**Keywords:** Hepatitis B virus, HBx, C-terminal deletion, Point mutations, Hepatocellular carcinoma, Genotype D, Structural modeling, HRM, Liver tissue

## Abstract

**Background:**

Hepatitis B virus (HBV) infection remains a major global cause of hepatocellular carcinoma (HCC), with the X protein (HBx) playing a central role in viral replication and host-virus interactions. Genetic variability within HBx, particularly in genotype D1, may influence disease progression through structural and functional alterations.

**Methods:**

In this study, the HBx region from an HBV-infected patient with HCC was analyzed using high-resolution melting (HRM) and direct sequencing. The obtained sequence was compared with a genotype D1 reference, followed by in silico structural interpretation.

**Results:**

Sequence analysis revealed a distinct mutational profile characterized by multiple amino acid substitutions within the transactivation domain, including H94R, S101S (synonymous), L116S, and V131T, along with a C-terminal truncation spanning amino acids 142–154 (C-terminal deletion/truncation). These alterations were located within or adjacent to functionally important regions, including domains implicated in transcriptional regulation, mitochondrial signaling, and protein-protein interactions. HRM analysis demonstrated a shift in melting temperature consistent with altered nucleotide composition. Structural interpretation suggested that the combined presence of point mutations and C-terminal truncation may contribute to localized conformational changes and altered interaction potential, although no definitive functional conclusions can be drawn.

**Conclusion:**

The identified HBx variant represents a complex mutational pattern involving both non-synonymous substitutions and a C-terminal truncation, which may reflect a multi-step process of structural and functional perturbations. This observation expands the current understanding of HBx variability in genotype D1 HBV and highlights the potential relevance of combined mutational profiles in the context of HBV-associated hepatocarcinogenesis. Further studies are required to clarify the biological significance of these findings.

## Graphical Abstract

**Figure:**
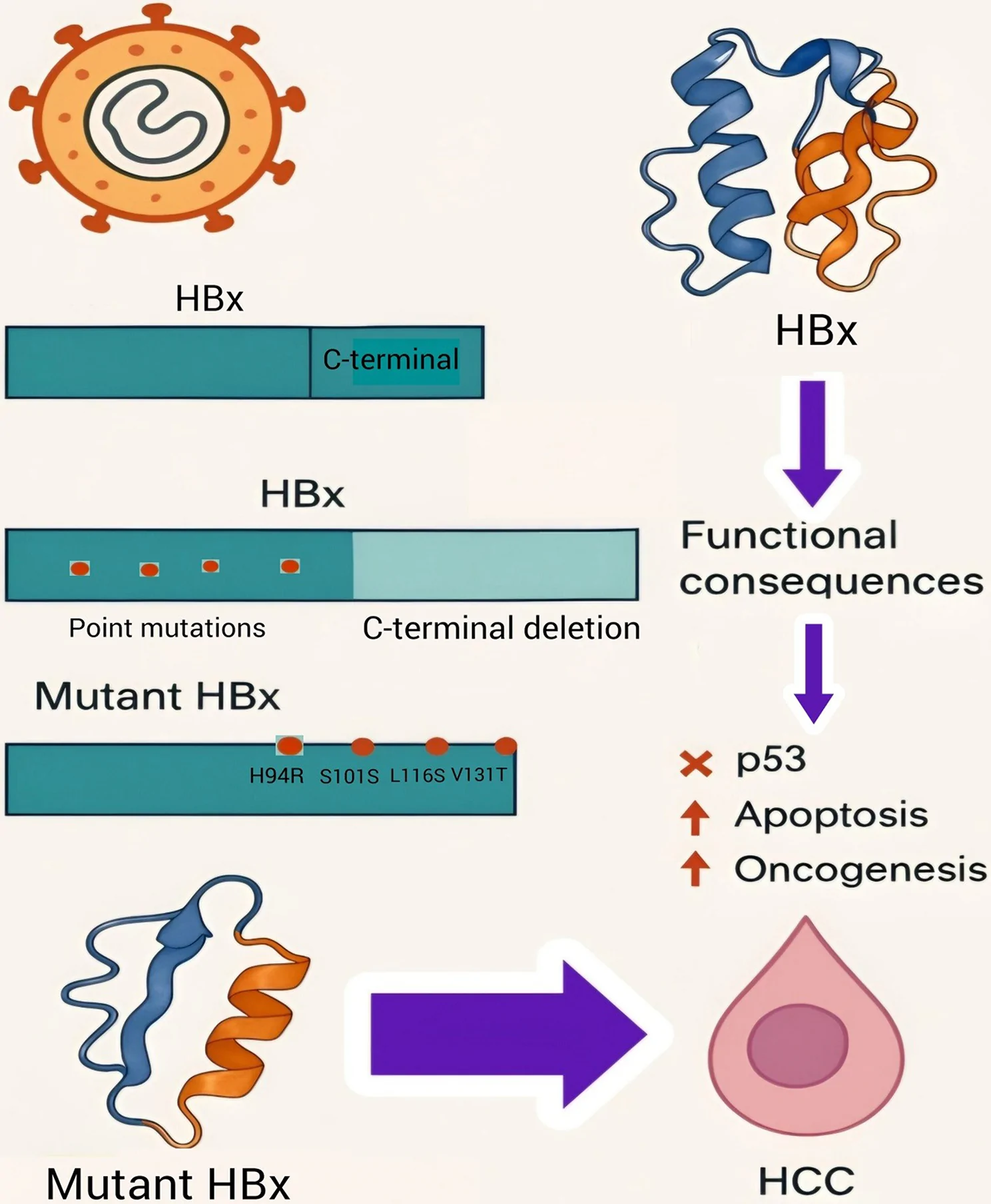

## Introduction

Hepatitis B virus (HBV) is a partially double-stranded DNA virus that infects over 250 million people worldwide and remains a major cause of chronic hepatitis, cirrhosis, and hepatocellular carcinoma (HCC). Among its four overlapping open reading frames, the HBx gene encodes a 154-amino-acid regulatory protein that plays essential roles in viral replication and hepatocarcinogenesis. HBx is a multifunctional transactivator that engages in a complex and multidirectional host interactome, modulating numerous cellular pathways, including epigenetic regulation, signal transduction, apoptosis, and immune evasion [1–3]. Notably, HBx interacts with a wide range of host factors and signaling cascades such as NF-kB, Wnt/β-catenin, and interferon-related pathways, thereby contributing to viral persistence and tumor development [2, 3]. The C-terminal region of HBx contains key functional elements, including nuclear localization signals, transactivation motifs, and interaction sites for host proteins such as p53, DDB1, and proteasome-associated factors. Genetic alterations in this region—particularly point mutations and partial deletions—can disrupt these interactions and modify HBx activity. Previous studies indicate that C-terminally truncated HBx variants may promote oncogenesis by impairing tumor-suppressive pathways, enhancing transcriptional deregulation, and contributing to genomic instability [4]. Deletions affecting the last 20–40 amino acids frequently emerge during long-standing chronic infection and may confer selective advantages related to immune escape or viral persistence [5]. Despite increasing recognition of the clinical relevance of C-terminal HBx mutations, the structural and functional consequences of such variants—especially when combined with concurrent point mutations—remain insufficiently characterized. In addition, recent findings from our previous study published in Scientific Reports [6] highlighted the presence of distinct mutational patterns within the HBx gene in chronic HBV infection, supporting the concept that HBx variability may reflect adaptive virus-host interactions. Here, we report a unique HBx variant identified in a patient with confirmed HCC, featuring a substantial C-terminal partial deletion accompanied by multiple adjacent point mutations. Using high-resolution melting (HRM) analysis, Sanger sequencing, and in silico structural modeling, we examined the potential molecular consequences of these alterations. This case highlights the importance of HBx structural integrity in HBV-associated carcinogenesis and underscores the value of computational modeling for interpreting clinically relevant HBV mutations.

## Case presentation

A 52-year-old man from Kerman, Iran, with a 20-year history of chronic HBV genotype D infection and inconsistent antiviral therapy, presented with progressive abdominal discomfort, fatigue, and unintentional weight loss. Abdominal ultrasonography and contrast-enhanced computed tomography (CT) demonstrated a large hepatic mass in the right lobe, highly suggestive of hepatocellular carcinoma (HCC). Serum AFP levels were markedly elevated (> 500 ng/mL). Virological assessment revealed a high HBV viral load (> 10⁶ IU/mL), with negative serology for HCV, HDV, and HIV. Liver function tests showed elevated ALT and AST, with mildly decreased albumin. Archived liver tissue samples, obtained post-mortem in 2022, were used for molecular analysis. Viral DNA extracted from the tissue underwent HRM screening, which demonstrated atypical melting curves suggestive of underlying genetic variation. Sanger sequencing subsequently confirmed a large partial deletion in the C-terminal region of HBx alongside multiple point mutations. Alignment with the HBV genotype D reference sequence (U95551.1) validated these findings. This case reveals a distinct HBx mutant associated with advanced HBV-related HCC and emphasizes the need for molecular characterization of tumor-derived viral sequences.

### Patient samples and DNA extraction

Archived liver tissue from the 52-year-old patient with HBV genotype D infection and advanced HCC was used for molecular characterization. Viral DNA was extracted using the QIAamp DNA Mini Kit (Qiagen, Hilden, Germany) according to the manufacturer’s protocol. DNA concentration and purity were assessed using a NanoDrop spectrophotometer (Thermo Fisher Scientific, Waltham, MA, USA), ensuring suitability for downstream PCR and sequencing analyses.

### PCR amplification and primer design

The HBx gene was amplified using high-fidelity Taq DNA polymerase (Thermo Fisher Scientific) in a conventional PCR platform. Cycling conditions were as follows: initial denaturation at 95 °C for 5 min; 35 cycles of denaturation at 95 °C for 30 s, annealing at 58 °C for 30 s, and extension at 72 °C for 1 min; followed by a final extension at 72 °C for 7 min. Two primer sets were designed to target conserved regions of HBx (Table 1). The first set generated a 168-bp fragment suitable for rapid screening using real-time PCR and HRM analysis. The second set amplified a 506-bp fragment encompassing the full coding region to enable Sanger sequencing and mutation profiling. Primer specificity was verified using Primer-BLAST (NCBI).

**Table 1:** Specific primer sequence for detection and sequencing of HBx

Name	Real-time PCR primers	Location	Product size (bp)
HBXF:	GTCTGTGCCTTCTCATCTG	265–284	168 bp*
HBXR:	GTTCACGGTGGTCTCCAT	433 − 415	
**Name**	**Sequencing Primers**	**Location**	
HBXSF:	ATGGCTGCTAGGCTGTGCT	-19-0	506 bp
HBXSR:	GGCAGAGGTGAAAAAGTTGC	487 − 465	

*The 168 bp product was used for initial HBx detection; the 506 bp product covered the full-length coding sequence and was used for mutation analysis

### High-resolution melting (HRM) analysis

HRM analysis was performed using the Rotor-Gene Q real-time PCR system (Qiagen) to screen for sequence variations within the HBx region. Melting curves obtained from each sample were compared with the wild-type control. Samples exhibiting reproducible deviations from the wild-type melting pattern were selected for downstream sequencing. HRM served as a rapid and sensitive preliminary screening tool to identify potential mutations.

### Sanger sequencing and mutation identification

PCR products showing atypical HRM transitions were purified using ExoSAP-IT (Thermo Fisher Scientific) and sequenced bidirectionally on an ABI 3500 Genetic Analyzer. Resulting chromatograms were quality-checked and aligned against the HBV genotype D reference sequence (GenBank accession no. U95551.1) using MEGA X and confirmed with NCBI BLAST. Both point mutations and a major C-terminal partial deletion were identified. Mutations were mapped relative to known HBx domains, with particular attention to the enhancer II region, transactivation motifs, and zinc-binding elements that are frequently implicated in hepatocarcinogenesis.

### Phylogenetic analysis

Multiple sequence alignment of the one HBx sequence with representative HBV genotypes retrieved from GenBank was performed using MUSCLE. A phylogenetic tree was constructed using the Maximum Likelihood (ML) method with the Tamura-Nei model and 1,000 bootstrap replications in MEGA X software.

### 3D protein modeling and structural analysis

The amino acid sequences of both wild-type and mutant HBx proteins were submitted to the Swiss-Model server (https://swissmodel.expasy.org) for in silico homology-based 3D modeling. The resulting models were visualized and analyzed using UCSF Chimera software (Resource for Biocomputing, Visualization, and Informatics, University of California, San Francisco, USA). Analyses included structural alignment, assessment of secondary structure elements, surface charge distribution, and domain organization. Special focus was placed on alterations within the C-terminal region, including a large deletion, to evaluate its potential impact on HBx transactivation function and DNA-binding capacity.

## Results

**Fig. 1:**
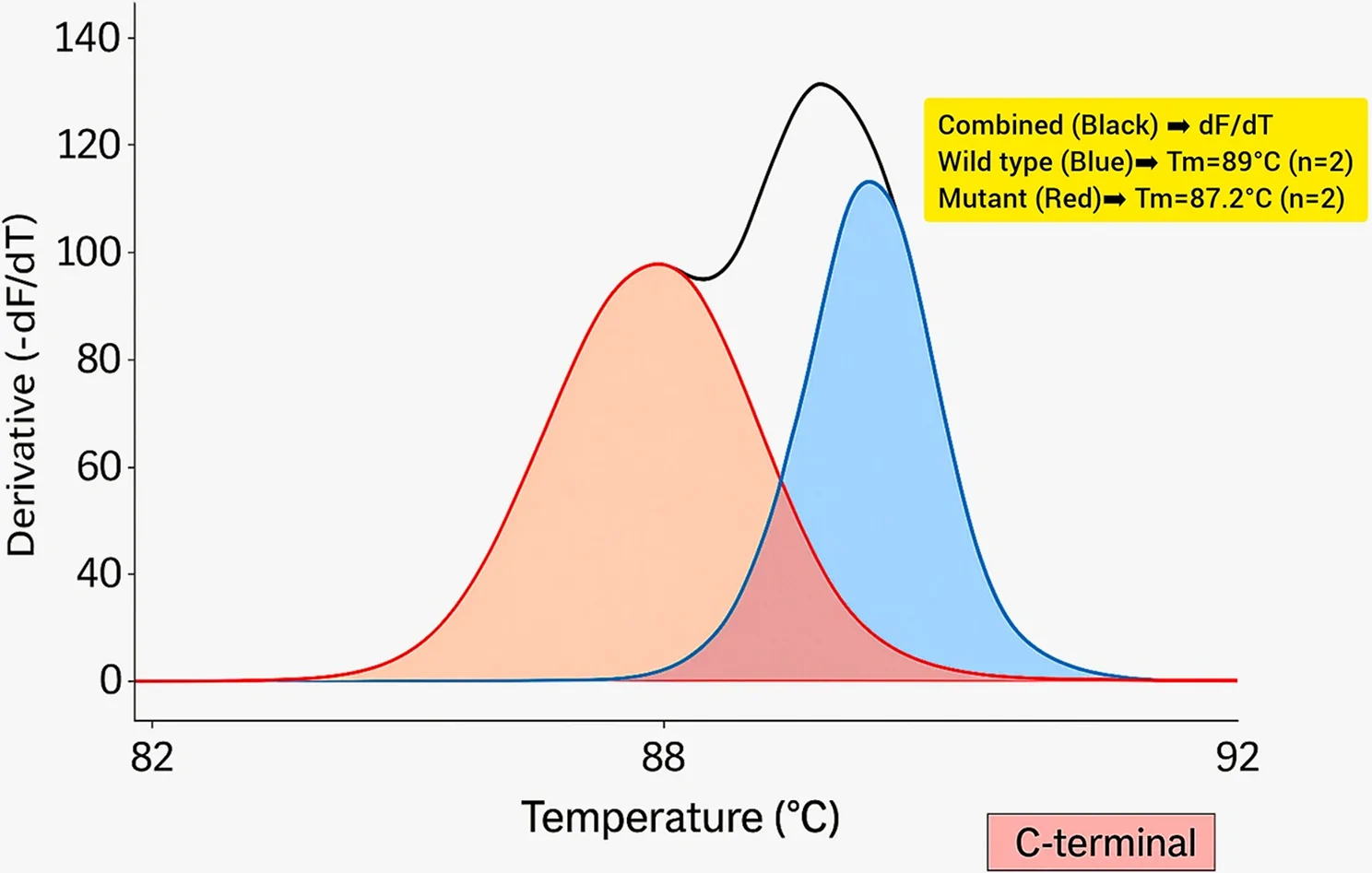
HRM analysis of HBx wild-type and mutant variants

HRM curve analysis clearly distinguished between the HBx wild-type and the C-terminal deletion mutant. As shown in Fig. 1, the wild-type exhibited a sharp melting peak at 89.0 °C, whereas the mutant variant demonstrated a reproducible left-shifted melting transition with a peak at 87.2 °C. This ~ 1.8 °C reduction in melting temperature reflects a consistent decrease in thermal stability of the deletion mutant compared with the wild-type sequence. The combined melting profile further confirmed the distinct separation between the two populations, with no evidence of overlapping transitions. These findings indicate that the C-terminal deletion induces a measurable alteration in DNA duplex stability, consistent with changes in nucleotide composition. As HRM analysis reflects differences at the DNA level, no direct conclusions regarding protein structure or functional interactions can be drawn from these results.

**Fig. 2:**
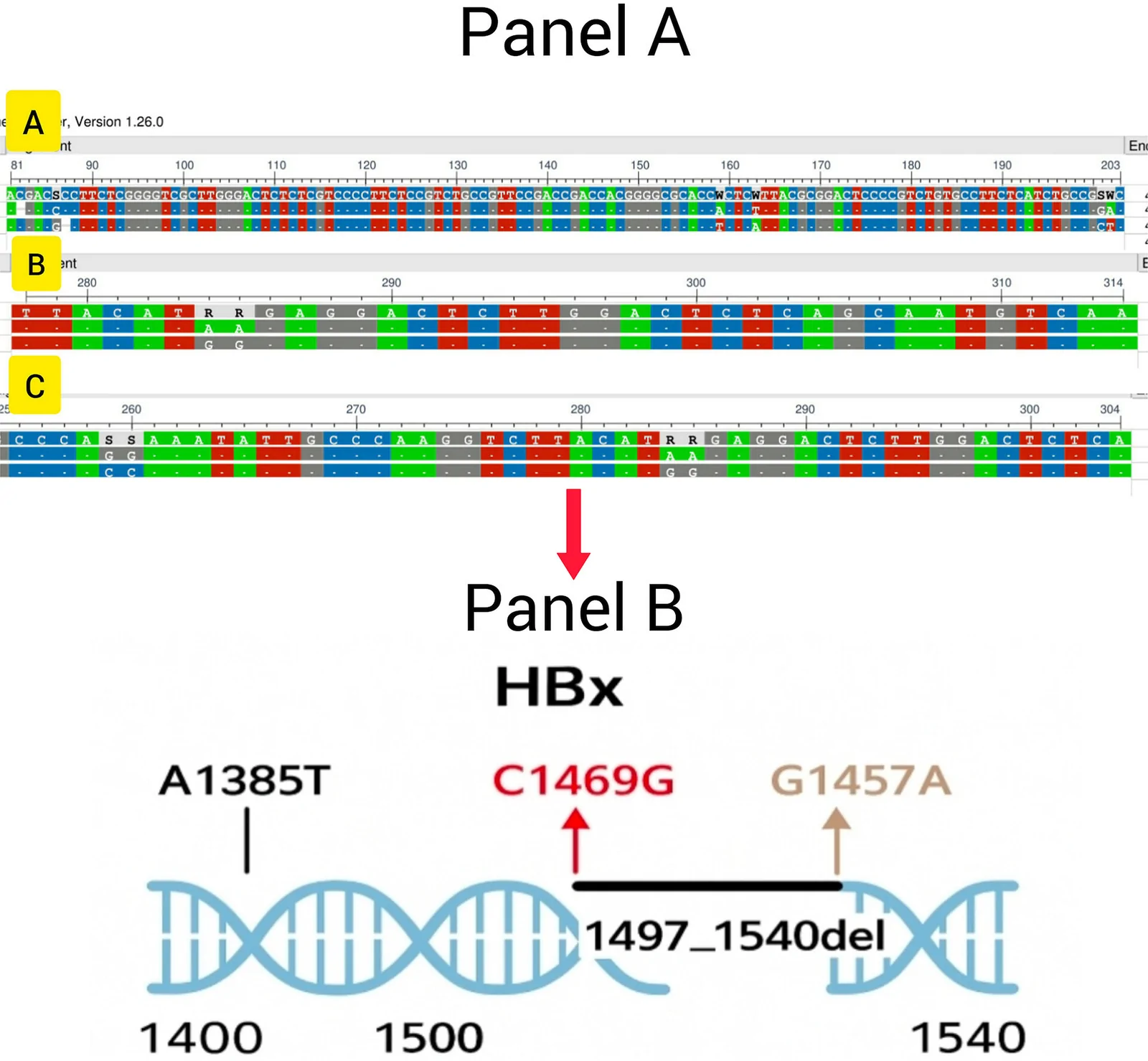
Integrated representation of HBx sequence alignment and mutation profile in genotype D1 HBV

Panel (A): Multiple sequence alignment of the HBx gene (Fig. 2) comparing the reference sequence (top) with the mutant sequence (bottom). The alignment is shown in segmented panels (A–C) to improve visualization and collectively represents the full-length HBx coding region. Several nucleotide substitutions are observed across the sequence. Gray dashes indicate deleted nucleotides, while colored residues represent mismatches relative to the reference sequence.

Panel (B): Schematic illustration of the identified mutations mapped onto the HBx genomic region (Fig. 2). Three point mutations (A1385T, G1457A, and C1469G) and a large deletion spanning nucleotides 1497–1540 (1497_1540del) are shown. The deletion corresponds to a C-terminal truncation of the HBx protein

Sequence alignment was performed using MEGA X and visualized in Geneious Prime. Comparative analysis was conducted using the NCBI BLAST platform against the HBV genotype D reference sequence (GenBank accession no. MT986034.1). The combined presence of point mutations and a C-terminal deletion highlights a complex mutational profile in the HBx gene.

**Fig. 3:**
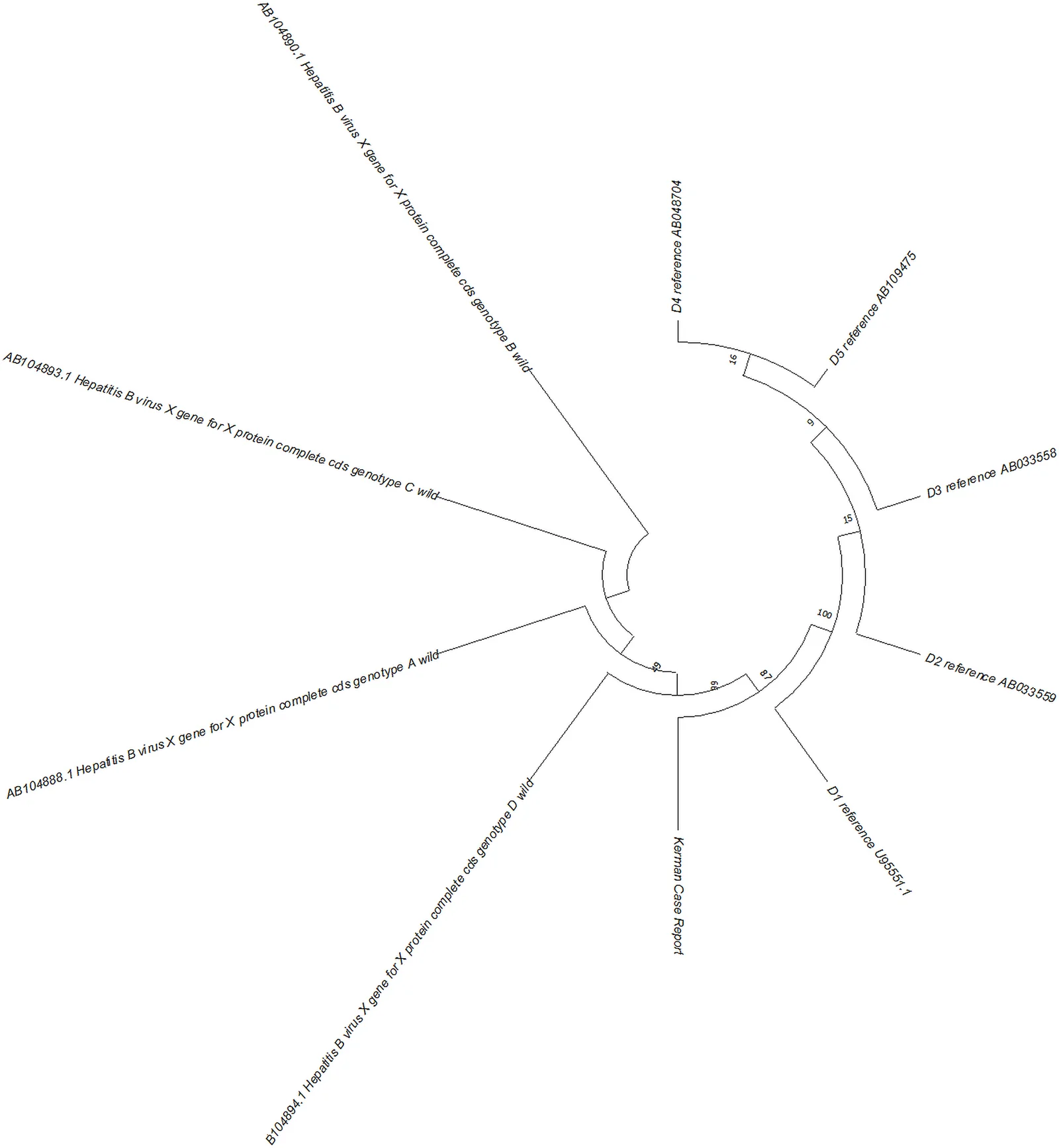
Phylogenetic tree of HBx gene sequences constructed using the Maximum Likelihood method in MEGA X. The Kerman Case Report isolate clusters within the genotype D1 clade, showing its closest relationship to reference sequences AB033559 and U95551.1. Evolutionary history was inferred using the Tamura–Nei substitution model, and the robustness of each branch was assessed with 1,000 bootstrap replications. Only bootstrap values ≥ 70% are shown. The tree was generated using aligned sequences (∼465 bp) after multiple sequence alignment with MUSCLE. The circular topology was selected for optimal visualization of phylogenetic clustering among HBV genotypes

To determine the evolutionary relationship of the HBx sequence (Fig. 3) obtained from the Kerman Case Report, a phylogenetic analysis was performed using MEGA X (version 10.x). All available reference sequences representing major HBV genotypes (A–D) and relevant subgenotypes were retrieved from GenBank and included in the analysis (*n* = 10 total sequences). Multiple sequence alignment was carried out using MUSCLE with default parameters. The final alignment contained approximately 465 nucleotides corresponding to the full-length HBx coding region. The evolutionary history was inferred using the Maximum Likelihood (ML) method, based on the Tamura–Nei model, which was identified as the best-fit substitution model according to the Bayesian Information Criterion (BIC). The reliability of the inferred tree topology was evaluated using 1,000 bootstrap replicates. Branches supported by bootstrap values ≥ 70% were considered statistically robust. The tree was visualized in circular layout, enabling clear separation of HBV genotypes and subgenotypes. The phylogenetic results demonstrated that the HBx sequence from our patient clusters tightly with subgenotype D1 reference sequences, including AB033559 and U95551.1, indicating that the Kerman Case Report isolate belongs to HBV genotype D1, which is the predominant circulating genotype in the Middle East and Iran. This clustering pattern is consistent with previously published molecular epidemiology data from the region.

**Table 2:** Protein-level characterization of HBx mutations identified in the present case compared to the reference genotype D1 sequence

No.	Position (aa)	Wild-type (Ref)	Mutant	Type of Mutation	Functional Region	Predicted Impact
1	94	H (Histidine)	R (Arginine)	Missense	H-box / DDB1-binding region	May alter host protein interaction
2	101	S (Serine)	T (Threonine)	Missense (conservative)	Transactivation domain	Minor structural modulation
3	116	L (Leucine)	S (Serine)	Missense (non-conservative)	Transactivation domain	Alters hydrophobicity, potential structural instability
4	131	V (Valine)	T (Threonine)	Missense	Near BH3-like region	May influence apoptotic signaling
5	**142–154**	**Present**	**Deleted**	**Truncation (C-terminal deletion)**	**C-terminal regulatory region**	**Potential loss of transcriptional activity and altered mitochondrial and oncogenic functions**

The Table 2 summarizes non-synonymous amino acid substitutions and the C-terminal truncation detected in the HBx protein of the studied isolate. Mutations are mapped according to their amino acid position relative to the reference sequence (Genotype D1), and are categorized based on mutation type, structural domain, and predicted functional relevance. The transactivation domain (aa 51–140), which plays a central role in host–virus interactions, contains the majority of observed substitutions, including both conservative (S101T) and non-conservative (L116S, V131T) changes. Notably, the H94R substitution is located within or proximal to the H-box/DDB1-binding region, a critical motif involved in ubiquitin-mediated regulatory processes. The most prominent alteration is a truncation event resulting in the deletion of the C-terminal segment (aa 142–154), a region previously implicated in transcriptional activation, mitochondrial localization, and modulation of apoptotic pathways. Functional region assignments are based on previously published studies of HBx structure-function relationships. Predicted impacts are inferred from amino acid properties and in silico interpretation and should be considered as putative.

Importantly, unlike certain previously reported HBx truncations occurring at glycine residues (e.g., G135), the truncation observed in this case does not directly involve a glycine position. Nevertheless, given the established role of glycine residues in maintaining structural flexibility and conformational adaptability, it is conceivable that alterations within or near glycine-rich regions may indirectly contribute to structural destabilization and facilitate truncation events. Overall, the combined presence of multiple point mutations and a C-terminal deletion highlights a complex mutational profile that may influence HBx functional dynamics and its role in HBV-associated hepatocarcinogenesis.

**Fig. 4:**
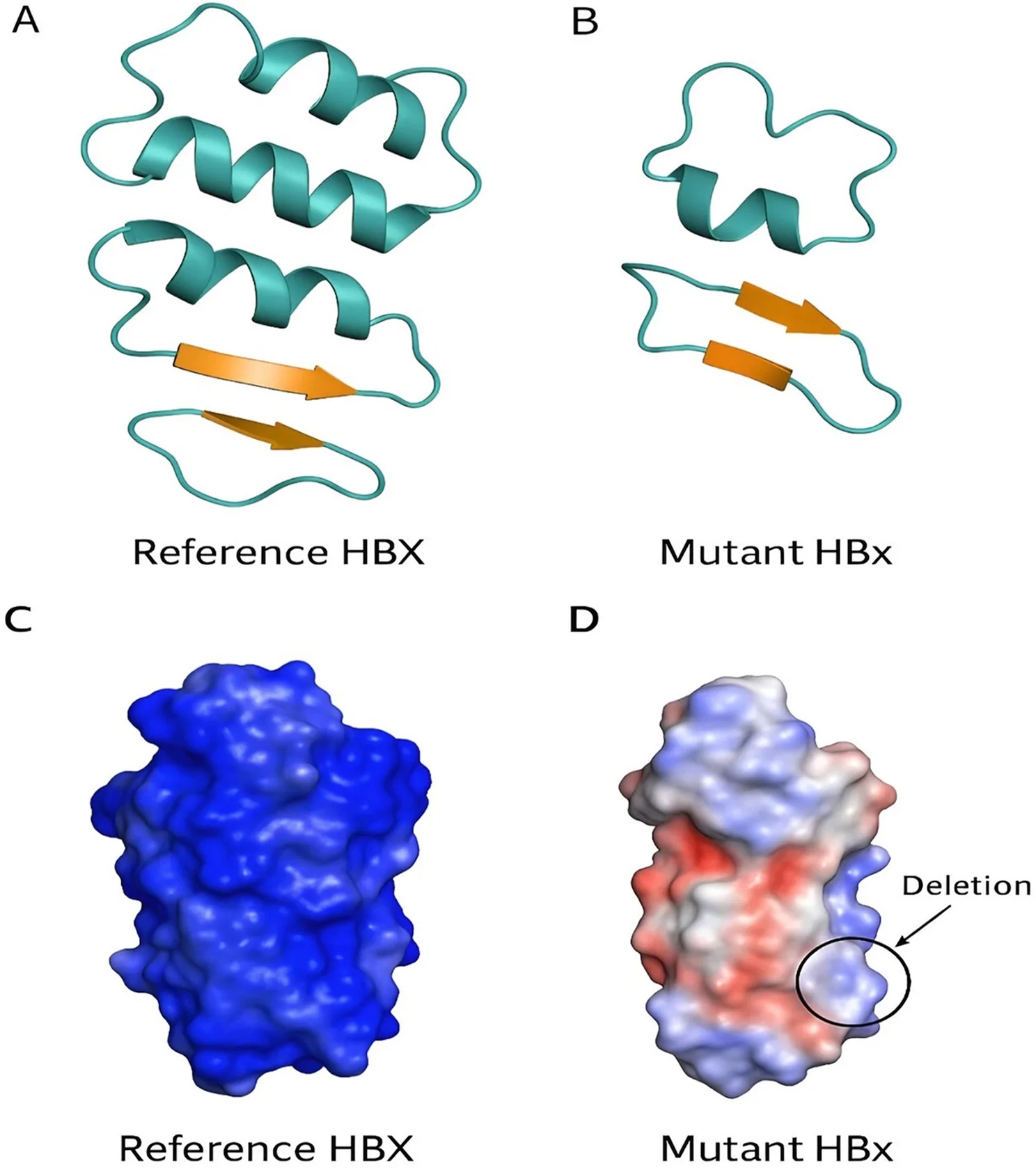
Structural comparison between reference and mutant HBx proteins

The comparative structural analysis of reference and mutant HBx proteins is presented in Fig. 4. This figure compares the predicted 3D conformations of the wild-type HBx protein and the mutant variant using homology models generated by SWISS-MODEL and visualized in UCSF Chimera. (A) The reference model displays four α-helices (cyan) and two β-sheets (orange), consistent with previously described HBx secondary-structure elements. (B) The mutant HBx protein containing a C-terminal deletion suggests structural alterations, including reduced α-helical content and localized changes in overall folding. (C) The surface rendering of the reference HBx model demonstrates a compact globular architecture. (D) The electrostatic surface map of the mutant HBx protein highlights a discrete gap at the deletion site (black circle) together with visible changes in surface charge distribution (red = negative, blue = positive). These in silico models suggest that the observed deletion and accompanying point mutations may influence the stability and surface topology of HBx, which could, in turn, affect its interaction interfaces. While these structural predictions offer useful insight, experimental validation will be required to determine the actual biological consequences of these alterations.

## Discussion

HBx is a multifunctional regulatory protein involved in viral replication, host-cell signaling, and HBV-associated hepatocarcinogenesis [7]. In this case, we identified a mutant HBx sequence containing a substantial C-terminal deletion (beginning at amino acid 142) along with several point mutations distributed throughout the open reading frame. The deleted segment includes regions previously associated with transcriptional activation, mitochondrial targeting, and apoptotic modulation. Alterations within this domain have been linked to changes in protein stability, subcellular localization, and regulatory activity [8]. Prior studies have also reported that C-terminally truncated HBx variants are more frequently detected in tumor tissues compared with adjacent non-tumorous liver, suggesting a possible association with hepatocarcinogenesis [9]. Truncation may also reduce the ability of HBx to interact with host tumor suppressors such as p53, potentially affecting apoptotic responses and genomic stability [10]. HRM analysis in our study revealed a reproducible melting temperature shift between wild-type and mutant HBx amplicons. The deletion mutant showed a lower Tm (87.2 °C) compared with the wild-type sequence (89.0 °C), indicating reduced duplex stability. Although HRM findings do not provide functional information, this difference is consistent with an altered nucleotide composition and potential changes in local folding dynamics. Protein-level alignment of the HBx sequence derived from the present case revealed a distinct mutational profile characterized by both multiple amino acid substitutions and a C-terminal truncation. The identified substitutions are predominantly located within the transactivation domain (aa 51–140), a functionally critical region mediating interactions with host transcriptional regulators, signaling molecules, and mitochondrial pathways. Among these, the H94R substitution is positioned within proximity to the H-box/DDB1-binding motif, suggesting a potential impact on HBx-mediated ubiquitin ligase recruitment and downstream regulatory processes [11, 12]. In addition, the L116S substitution represents a non-conservative amino acid change involving a shift from a hydrophobic to a polar residue, which may influence local folding dynamics or protein stability. Similarly, the V131T substitution occurs near the BH3-like region, a segment previously implicated in apoptotic signaling modulation, raising the possibility of altered host–cell interaction patterns. Although the S101T substitution is conservative in nature, its occurrence within a regulatory domain suggests that even subtle physicochemical changes may contribute to cumulative functional effects [6, 13, 14]. The most notable finding is the truncation of the HBx protein at amino acid position 141, resulting in the loss of the C-terminal region (aa 142–154). This segment has been associated with transcriptional activation, mitochondrial targeting, and oncogenic signaling pathways, and its deletion may therefore lead to significant alterations in HBx functional capacity [15]. The functional regions of HBx were annotated based on previously reported domain organization and interaction studies [7, 16–18]. Interestingly, in contrast to previously described truncation events involving glycine residues—such as the G135 premature stop codon reported in HBV-associated HCC—the truncation observed in this case does not directly occur at a glycine position [19]. However, considering the intrinsic structural flexibility conferred by glycine residues within HBx [6], it is plausible that local conformational instability in glycine-rich regions may predispose the protein to truncating mutations, even when the truncation site itself is not located at a glycine residue. Structural modeling was performed for comparative purposes to evaluate potential differences between the reference and mutant HBx sequences. Given the lack of a full-length experimentally resolved structure and the presence of intrinsically disordered regions, the results should be interpreted with caution. Recent advances in protein structure prediction, such as AlphaFold, further support the presence of both structured and disordered regions within HBx. Collectively, these findings suggest that the interplay between distributed point mutations and C-terminal truncation may contribute to altered HBx structural integrity and functional behavior, providing further insight into the heterogeneous mutational landscape of HBx in genotype D1 HBV and its potential role in hepatocarcinogenesis.

## Limitations

This study has several limitations that should be considered when interpreting the findings. First, the analysis is based on a single patient sample, which restricts the ability to generalize the observed mutational pattern or infer causal relationships with clinical outcomes. Second, although HRM analysis, Sanger sequencing, and in silico structural modeling provide valuable preliminary insights, these approaches cannot replace functional assays. The predicted effects on protein folding, host–protein interactions, and downstream signaling pathways require experimental validation through cellular, biochemical, or virological studies. Third, the use of archived liver tissue may introduce technical constraints, including potential DNA degradation, which could influence sequencing depth or mutation detectability. Fourth, due to the compact and overlapping nature of the HBV genome, some mutations—particularly synonymous substitutions—may exert functional consequences in other reading frames, such as the polymerase gene. These potential effects were not evaluated in this study. Finally, homology-based 3D modeling has inherent limitations, particularly in the absence of a full-length experimentally resolved HBx structure. In addition, the intrinsically disordered nature of the N-terminal region may further reduce modeling accuracy and limit the reliability of structural interpretations in these regions. Despite these limitations, the present case provides a detailed structural and genomic characterization of a rare HBx variant and offers hypotheses that can guide future mechanistic and clinical studies. In addition, due to the PCR-based amplification strategy used in this study, it was not possible to determine whether the identified HBx sequence originated from episomal HBV DNA or from viral DNA integrated into the host genome. Accordingly, the presence of potential HBV-host fusion events could not be assessed. Given the known role of HBV integration in hepatocellular carcinoma, this represents an important limitation that should be addressed in future studies using genome-wide or transcriptome-based approaches.

## Conclusion

In conclusion, the HBx variant identified in this case is characterized by a distinct mutational profile involving the coexistence of multiple amino acid substitutions within the transactivation domain and a C-terminal truncation. This combined alteration pattern may reflect a multi-step process of structural and structural and functional perturbations, rather than a single mutation-driven effect. The distribution of mutations across key regulatory regions, together with the loss of the C-terminal segment, is consistent with potential alterations in HBx interaction capacity and signaling behavior. Although the precise functional consequences cannot be determined within the scope of this study, the observed pattern may suggest a form of progressive molecular adaptation during disease evolution. Importantly, this case highlights the complexity of HBx mutational landscapes in genotype D1 HBV and underscores the need for further investigations integrating structural, functional, and clinical data. Future studies involving larger cohorts and experimental validation will be essential to clarify the biological significance of such combined mutational profiles in HBV-associated hepatocarcinogenesis.

## Data Availability

No datasets were generated or analysed during the current study.
